# Targeting the metabolic niche: vulnerabilities and opportunities in brain metastases

**DOI:** 10.1093/oons/kvag016

**Published:** 2026-10-01

**Authors:** Anthony G Chesebro, Charles D Kocher, Daniel Grange, Joseph O Deasy, Corey Weistuch

**Affiliations:** Laufer Center for Physical and Quantitative Biology, Stony Brook University, Stony Brook, NY 11794, United States; Department of Medical Physics, Memorial Sloan Kettering Cancer Center, New York, NY 10065, United States; Department of Medical Physics, Memorial Sloan Kettering Cancer Center, New York, NY 10065, United States; Department of Medical Physics, Memorial Sloan Kettering Cancer Center, New York, NY 10065, United States; Department of Medical Physics, Memorial Sloan Kettering Cancer Center, New York, NY 10065, United States

**Keywords:** brain metastasis, tumor microenvironment, metabolic niche, ketogenic diet

## Abstract

Brain metastases are the most common intracranial tumor in adults, and most arise from lung, breast, or melanoma. We propose a conceptual framework organized around three requirements for colonization, each unevenly distributed across the brain: vascular access, metabolic substrate, and escape from immune surveillance. We organize the literature around this three-axis framework and argue that metabolism, though the least exploited clinically, is where personalization may deliver the largest near-term gains. We review how lung, breast, and melanoma metastases each pair a characteristic metabolic program with characteristic anatomical distributions. Building on this, we discuss how targeted metabolic therapy, primary cancer-informed prediction, and longitudinal modeling can bring metabolism into precision brain metastasis care.

## Introduction

Brain metastases are the most common intracranial tumor in adults, and their incidence is rising as systemic disease control improves and routine MRI surveillance increases detection (Franchino et al. 2018, Mills et al. 2022). Three primary cancers (lung, breast, and melanoma) together account for the substantial majority of brain metastases, but the *where* and the *why* of brain metastasis differ greatly among them. For example, triple-negative breast cancer favors frontal and limbic cortex, while HER2+ disease seeds the occipital lobe and cerebellum (Kyeong et al. 2017). Non-small cell lung cancer (NSCLC) tends toward parieto-occipital cortex and the cerebellum (Quattrocchi et al. 2012), and small cell lung cancer (SCLC) concentrates in cerebellum and deep white matter (Shi et al. 2023). Melanoma preferentially settles in frontal cortex, with markedly less affinity for the cerebellum compared with the other primary cancers (Schroeder et al. 2020, Barrios et al. 2025). An atlas of >13 000 lesions confirmed earlier findings (Hwang et al. 1996) that metastases concentrate near the gray-white matter junction and watershed vascular territories. Deviations from that baseline (i.e. less reliance on local vascular supply) are tumor-specific and can potentially drive metastatic potential (Barrios et al. 2025).

We propose that the requirements for a tumor cell to survive in the brain can be organized around three axes. It requires (i) access to a vascular supply through which it can also penetrate the blood-brain barrier (BBB) (García-Gómez and Valiente 2020), (ii) abundant metabolic substrate (Celià-Terrassa and Kang 2018), and (iii) the ability to evade or co-opt local immune surveillance (Schulz et al. 2019). Vascular caliber (Barrios et al. 2025), baseline glucose metabolism (Herculano-Houzel 2014), glia-to-neuron ratio (Tan et al. 2020), astrocyte and microglial transcriptional state (Tan et al. 2020, Stoessel and Majewska 2021), and even ketone body transporter expression (Leino et al. 2001) all vary by brain region. This regional heterogeneity suggests that tumor cells that arrive at a region whose vascular, metabolic, and immune properties match the cell’s intrinsic program or an ability to adapt to these conditions are the ones that establish lesions. As systemic therapies grow more effective at clearing extracranial disease, the BBB shelters intracranial tumor cells from drug exposure, and the central nervous system becomes a disproportionate site of relapse and death (Sperduto et al. 2017). The brain may therefore be enriched for cells that survived systemic treatment, which makes understanding what the brain environment selects for, metabolically and otherwise, a question of direct therapeutic consequence.

Of the three axes, metabolism is the least clinically investigated and possibly the most open to intervention, since many metabolic manipulations are inexpensive and already available. We take it as our primary focus for that reason, but not because we take it to be the dominant determinant of where metastases arise. Most current systemic therapies target signaling (HER2, BRAF/MEK, EGFR, and ALK) or immune checkpoints, and most local therapies are blind to the metabolic state of the lesion they treat (Soffietti et al. 2020). Yet evidence is accumulating that metastases in different brain regions differ in glycolytic dependence (Bao et al. 2024), lipid handling (Ferraro et al. 2021), glutamine reliance (Fischer et al. 2021), and lactate biology (Yi et al. 2025), and that these differences are exploitable. They also suggest that uniform dietary or metabolic interventions may produce opposite effects depending on tumor genotype and lesion location. Notably, the ketogenic diet, which is currently in phase 2 trials (NCT05708352) for glioblastoma (Amaral et al. 2024), is attractive in brain metastasis because ketone bodies cross the BBB on the same monocarboxylate transporters that move lactate. Ketosis is also independently anticonvulsant in a setting where seizures are a major cause of morbidity and mortality (Zheng et al. 2022).

In this perspective, we organize the literature around the proposed three axis framework. We first describe the universal vascular, metabolic, and immune requirements of a brain metastatic niche, and how regional heterogeneity in normal brain creates differential “soils” for these requirements (Section Requirements of the metastatic niche, Fig. 1). We then discuss the three principal primary cancers (breast, lung, and melanoma), describing how each primary cancer’s typical metabolic phenotype interacts with its preferred anatomical distribution (Section Metabolism and metastatic primary sources). Section Therapeutic interventions: state of the art and pitfalls reviews the current standard of care for brain metastases through the same three axis lens, identifies where current therapy is blind to metabolism, and uses the divergent literature on the ketogenic diet in melanoma as a worked example of why blanket metabolic intervention is likely to fail. We conclude by proposing what targeted metabolic therapy, primary-tumor-informed prediction, and longitudinal metabolic modeling would need to look like to bring metabolism into precision brain metastasis care (Section The future of targeted metabolic therapeutics, Fig. 2).

**Figure 1:**
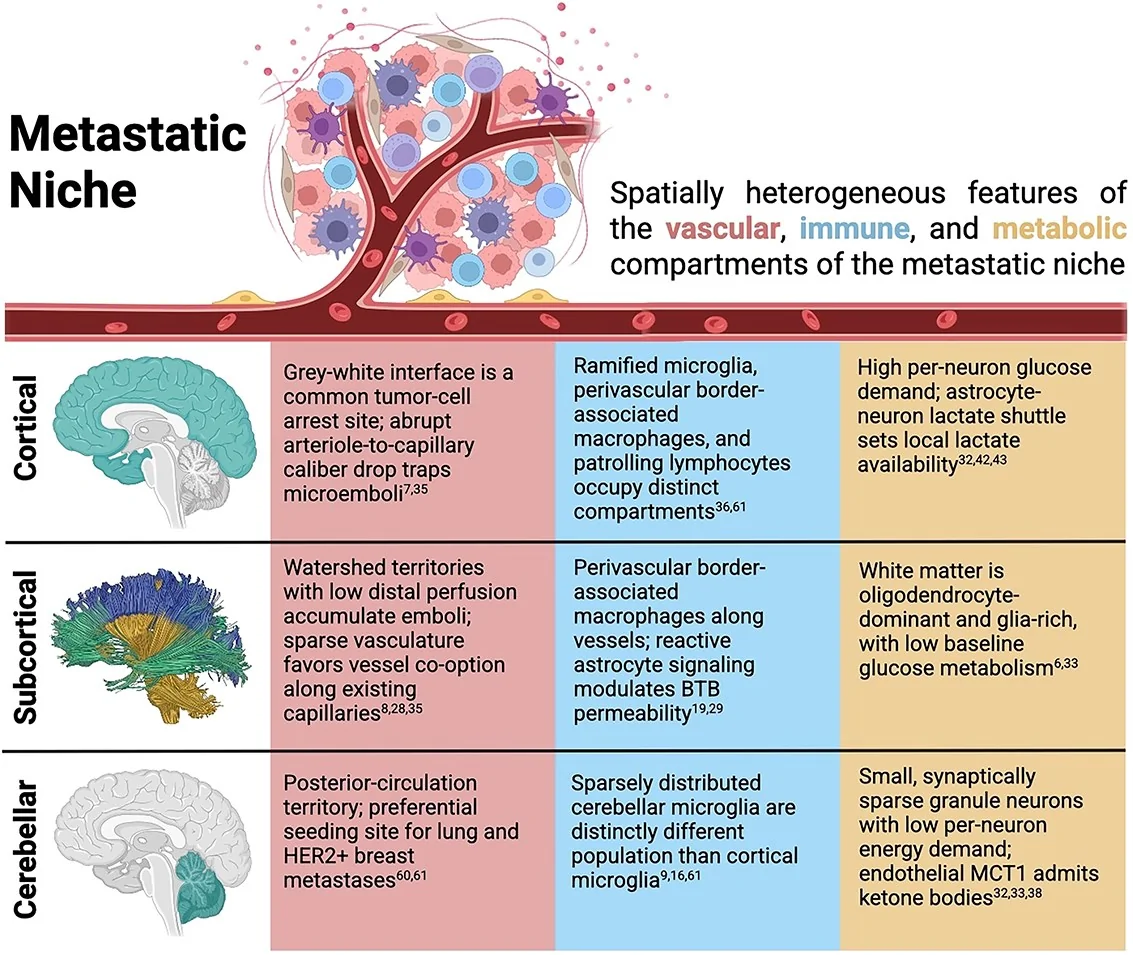
Proposed regional heterogeneity of the brain metastatic niche across three requirements for colonization. We propose that establishing a lesion requires vascular access, metabolic substrate, and escape from immune surveillance, and that each of these axes (columns) is nonuniformly distributed across brain regions (rows). The cortex, white matter and watershed territories, and cerebellum differ in vascular architecture, baseline metabolism and cellular composition, and resident immune populations, such that a tumor cell arriving in one region encounters a different environment than the same cell arriving in another. Regional placement of each feature reflects where the process is most salient. As detailed in the cited references, many features are found to varying degrees in all locations. Created with BioRender.com.

**Figure 2:**
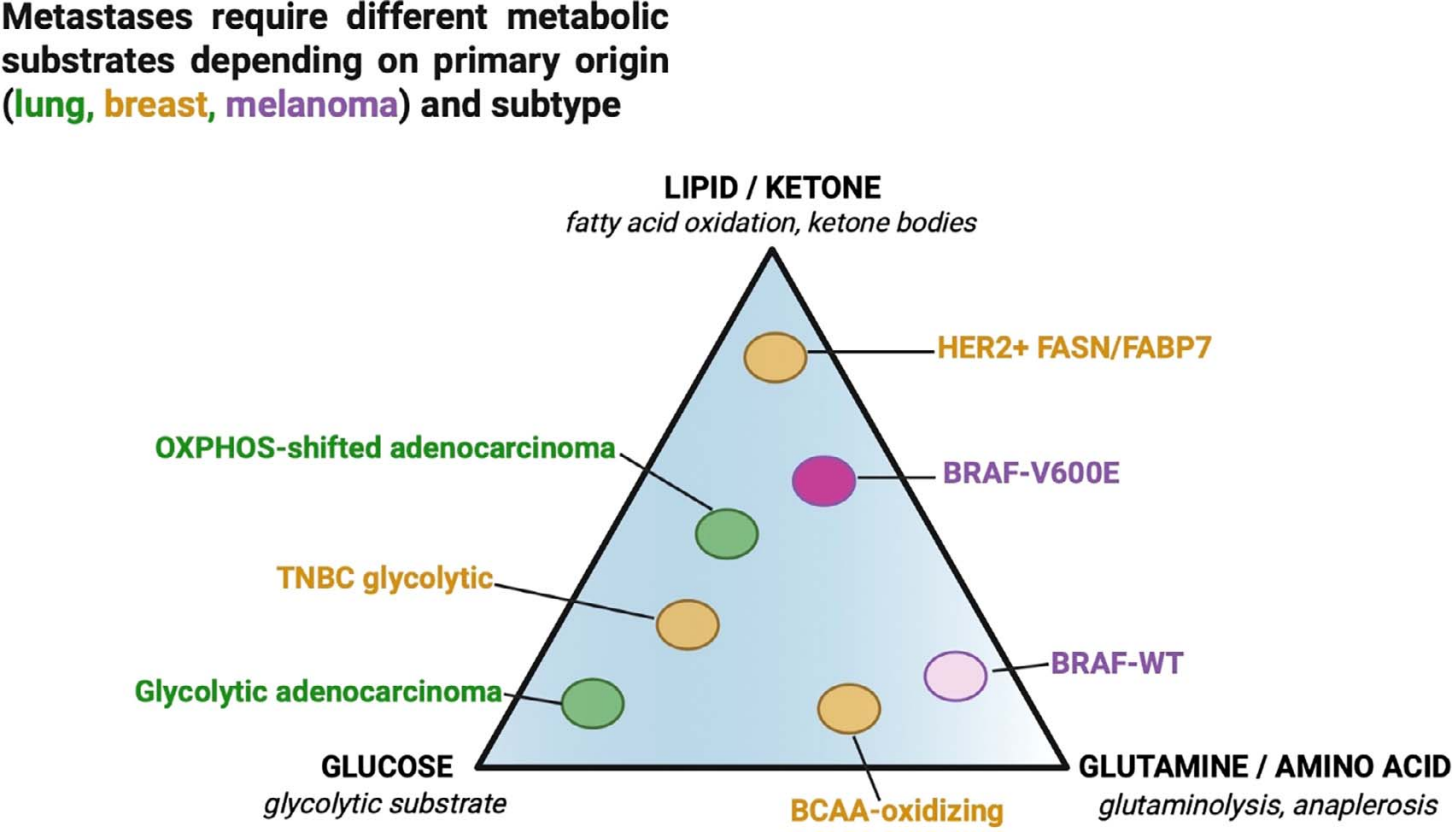
A substrate-reliance framework for brain metastases, positioned by tumor type and metabolic phenotype. Each vertex represents reliance on one carbon source: glucose (glycolysis), lipid and ketone metabolism, or glutamine and amino acids. Tumor types are placed according to the substrate evidence cited for each, with positions intended as qualitative summaries of that evidence rather than quantitative coordinates. Melanoma brain metastases are glutamine-leaning, reflecting their oxidative phosphorylation and glutaminolysis dependence (Fischer et al. 2021), with BRAF V600E disease shifted toward the ketone axis through acetoacetate-driven signaling (Xia et al. 2017). Lung adenocarcinoma spans a glycolytic to oxidative range, capturing its metabolic plasticity: a glycolytic, lactate-producing phenotype at one end (Duan et al. 2024, Yi et al. 2025) and an oxidative phosphorylation-activated phenotype at the other (Faubert et al. 2017, Duan et al. 2024). Breast metastases separate by subtype, with HER2+ disease relying on *de novo* fatty acid synthesis (Cordero et al. 2019, Ferraro et al. 2021) and TNBC engaging a core glycolytic program (Yoo et al. 2025). Proposed interventions act by shifting a tumor away from its preferred substrate or by targeting the dependency of the niche it occupies; their evidence ranges from preclinical to principle transferred from other cancers, and the ketogenic diet illustrates the central caution of the framework, suppressing BRAF wild-type melanoma while potentially feeding BRAF V600E disease (Xia et al. 2017). Created with BioRender.com.

## Requirements of the metastatic niche

### Vascular access and structure

Brain metastases preferentially locate near the gray-white matter junction and in watershed areas with rich vascular supply (Hwang et al. 1996, Barrios et al. 2025). Branching arterioles narrow abruptly as they cross from cortical gray matter into the more sparsely vascularized white matter, and tumor microemboli arrest at the caliber transition. Once seeded, tumor cells face a second vascular choice. They can induce sprouting angiogenesis, which produces leaky, permeable neovasculature visible on perfusion MRI, or they can co-opt existing vessels, growing along the abluminal surface of pre-existing capillaries without inducing new vessel formation (Bentolila et al. 2016). Vascular co-option is mediated in part by *β*1 integrins and L1CAM-mediated adherence to endothelial laminin (Valiente et al. 2014). Angiogenic metastases enhance avidly and respond, albeit transiently, to anti-VEGF therapy, while co-opting lesions show less robust enhancement, may resist anti-angiogenic agents (García-Gómez and Valiente 2020), and (as is frequently seen in melanoma (Bentolila et al. 2016)) predispose to hemorrhage as tumor cells weaken the walls of vessels they parasitize. A practical consequence of this vascular heterogeneity is the heterogeneity of the blood-tumor barrier (BTB) itself. BTB permeability can vary significantly between lesions within a single patient, and high permeability does not predictably track with lesion size or growth pattern (Lockman et al. 2010). Drug uptake is therefore inherently lesion-by-lesion, and systemic interventions that fail at one lesion may still be active at another.

### Immune and regulatory evasion

The classical view of the brain as immune privileged has given way to a more nuanced picture in which immune surveillance is regionally and compartmentally organized. Parenchymal microglia, perivascular and meningeal border-associated macrophages, choroid plexus macrophages, and patrolling lymphocytes each occupy distinct niches and show distinct transcriptional states (Klemm et al. 2020, Tan et al. 2020). Microglial transcriptional state varies regionally even in the steady-state adult brain, with cerebellar and cortical microglia showing different cytokine production profiles and different responses to injury (Tan et al. 2020). The astrocyte compartment is similarly plastic. Reactive astrocytes surrounding established brain metastases secrete TGF-*β*2 and CXCL10-related signals that modulate BTB permeability and immune cell infiltration, and the magnitude of these responses differs by tumor type and by anatomical site (Doron et al. 2019, Gong et al. 2019). Interacting with this regional immune architecture is the metabolic immunology of the tumor itself. Tumor-derived lactate can suppress NK-cell and CD8+ T-cell cytotoxicity (Brand et al. 2016), drive M2 polarization of tumor-associated macrophages and stabilize HIF1*α* (Colegio et al. 2014). Emerging evidence indicates that lactate-driven histone lactylation reprograms gene expression in both tumor and immune cells to entrench immunosuppression (Brand et al. 2016, Chen et al. 2024). Because lactate concentrations and clearance rates differ across brain regions as a function of normal astrocytic glycolysis and MCT distribution, the same tumor cell secreting the same amount of lactate will produce different degrees of local immunosuppression depending on where in the brain it seeds. The implication is that immune evasion in the brain is not a fixed property of the tumor, but an emergent one: the same metastatic cell will be more or less visible to immune clearance depending on the metabolic and immune characteristics of the region.

### Metabolic niche and substrate availability

There is significant regional heterogeneity in the metabolic requirements of healthy brain parenchyma. The average cortical neuron consumes *∼*20 times more glucose per minute than the average cerebellar neuron, despite the cerebellum housing the majority of the brain’s neurons (Herculano-Houzel 2011). Cerebellar granule neurons are small and synaptically sparse, so their per-neuron energy demand is intrinsically low (Herculano-Houzel 2011). Similarly, regional energy budgets appear to be optimized to minimize the cost of synthesizing and transporting the enzymatic machinery of metabolism (Gustafsson et al. 2024). The glia to neuron ratio inverts between cortex and cerebellum, with *∼*11 glia per neuron in supratentorial regions versus *∼*0.2 in the cerebellum (Herculano-Houzel 2014). Regional cell type composition affects extracellular lactate availability, glucose flux, and the dynamic range of the astrocyte-neuron lactate shuttle, as astrocytes are the principal site of brain glycolysis and neurons are net lactate consumers (Mason 2017, Magistretti and Allaman 2018). Ketone bodies and lactate cross the BBB on the MCT1 transporter. MCT1 expression is regionally heterogeneous and is upregulated by fasting and ketosis, meaning the same metastasis can have different access to circulating *β*-hydroxybutyrate depending on its location and the patient’s systemic metabolic state (Leino et al. 2001). Population-scale imaging studies confirm these regional differences are associated with different metastatic distributions. A probabilistic mapping study of 177 breast cancer patients showed brain metastases were significantly more likely to occur in regions of *lower* baseline glucose metabolism on healthy fluorodeoxyglucose-PET (FDG-PET) maps, accompanied by reduced expression of glycolysis and oxidative phosphorylation genes in these regions (Bao et al. 2024). The same atlas-scale approach in 548 lung cancer brain metastases found the opposite signal: lung lesions favored regions of higher baseline metabolic activity (Bao et al. 2024). We note that these associations should be interpreted bearing in mind that regional glucose metabolism covaries with vascular density, neuronal and glial composition, and resident immune populations. Separating these effects would require lesion-level rather than region-average measurement, and joint modeling of metabolic maps with perfusion and tissue-composition covariates. However, a plausible interpretation remains that primary origin and tumor subtype can therefore yield a distinct set of metabolic requirements, leading to a characteristic preferential metastatic distribution within the brain.

The vascular, metabolic, and immune axes are therefore not independent. They are coupled through the same cellular substrate (endothelium, astrocytes, microglia, and tumor cells), and they vary together across the brain. The driving factor for regional distributions is not clearly a single axis, and can incorporate other factors such as mechanical arrest at the gray-white matter junction where vessels become smaller (Hwang et al. 1996, Barrios et al. 2025). The next section examines how the three principal primary tumors exploit this coupling.

## Metabolism and metastatic primary sources

### Breast cancer

Breast cancer brain metastases (BCBM) can be divided into three broad categories. The pooled cumulative incidence is *∼*31% for HER2+ disease, 26% for triple-negative disease (TNBC), and 42% for HR+/HER2- disease (Mills et al. 2022). Spatial distribution differs by subtype: TNBCBM enrich in frontal and parietal cortex, while HER2+ lesions favor the occipital lobe and cerebellum, suggesting that subtype-specific systemic dissemination and local fit interact (Kyeong et al. 2017). At population scale, BCBM preferentially seed regions of lower baseline glucose metabolism and reduced metabolism-related gene expression, consistent with a “seed-and-soil” interpretation in which tumor-intrinsic metabolic programs are advantaged in regions where neither resident neurons nor astrocytes are themselves heavily glycolytic (Bao et al. 2024).

The intrinsic metabolic phenotype of brain-tropic breast cancer is reshaped to fit the lipid- and glucose-poor brain microenvironment. The most striking BCBM-specific metabolic adaptation reported to date is upregulation of *de novo* fatty acid synthesis. HER2+ breast cancer cells growing intracranially upregulate fatty acid synthase (FASN), and genetic or pharmacologic FASN inhibition reduces brain tumor growth, demonstrating a site-specific metabolic dependency that does not appear at the mammary fat pad (Ferraro et al. 2021). One possible basis is that the brain is relatively lipid-poor, as the BBB restricts entry of circulating fatty acids and lipoprotein particles, and CNS lipid is largely synthesized locally, so a metastasis that cannot import lipid must make it (Song et al. 2025). This makes FASN one of the few candidate targets in brain metastasis whose dependency is expected to be intracranially selective, and therefore one whose therapeutic window derives from the site rather than the tumor. Fatty acid binding protein 7 (FABP7) is similarly elevated in HER2+ BCBM relative to parental cells and correlates with worse survival (Cordero et al. 2019). Spatial transcriptomics of TNBCBM also identifies a core glycolysis transcriptional program and a neuron–tumor interaction signature at the tumor margin, indicating that BCBM cells engage with neuronal substrate availability at the lesion edge (Yoo et al. 2025).

Whether BCBM use ketone bodies as fuel is undetermined. The two-compartment stromal-ketogenesis model, in which stromal cells express HMGCS2, HMGCL, and BDH1 and export *β*-hydroxybutyrate and lactate that adjacent epithelial cancer cells import via MCT1, was developed in primary breast cancer co-culture systems and has not been directly tested in BCBM tissue (Martinez-Outschoorn et al. 2012). Definitive measurement of ketone utilization enzymes and flux in established BCBM lesions remains a gap. Perhaps the most provocative observation in this space is that brain adapted breast metastatic cells can grow glucose independently by oxidizing glutamine and branched chain amino acids, a phenotype that would, by definition, resist therapeutic strategies based purely on glucose restriction (Chen et al. 2015). We return to this idea of metabolic plasticity as the central barrier to substrate-directed intervention in Section Predicting brain metastatic metabolic phenotype from the primary tumor and first metastasis.

### Lung cancer

Lung cancer is the most common origin of brain metastases overall (Sperduto et al. 2017). NSCLC lesions are preferentially distributed in parieto-occipital cortex and cerebellum, with a substantial infratentorial fraction (Quattrocchi et al. 2012). SCLC carries one of the highest brain metastatic risks of any cancer, with >10% of patients presenting with intracranial disease at diagnosis (Steindl et al. 2019). A recent multicenter MRI mapping study found that SCLC and NSCLC both concentrate in deep white matter and cerebellar hemispheres, with SCLC showing a more diffuse and deeper distribution than NSCLC (Shi et al. 2023). An atlas-scale analysis additionally found that lung cancer brain metastases as a class favor regions of higher baseline metabolic activity, the opposite signal from BCBM and a direct empirical demonstration that “metabolic soil” is primary tumor-specific (Bao et al. 2024).

The metabolic phenotype of lung cancer brain metastases is plastic and nonuniform. In a lung adenocarcinoma brain metastasis model, AKR1B10-dependent metabolic reprogramming produced a hyperactive Warburg phenotype with elevated lactate and increased LDHA expression (Duan et al. 2024). Lactate production promoted histone lactylation, coupling glycolysis to chemoresistance through epigenetic reprogramming (Duan et al. 2024). Single-cell transcriptomic data across early primary, advanced primary, and brain-metastatic lung adenocarcinoma similarly identify glycolysis as a dominant tumor-cell program in metastasis, with reduced immune signaling and an associated immunosuppressive microenvironment dominated by M2 macrophages in high-glycolysis environments (Yi et al. 2025). Despite this evidence, the picture is not uniformly glycolytic. A multi-omics analysis of paired primary lung cancer and lung cancer brain metastases revealed mitochondrial-specific metabolic activation in the microenvironment and that oxidative phosphorylation inhibition was therapeutically effective in patient-derived organoids and *in vivo*, implying a subset of lung metastases shift toward oxidative metabolism (Duan et al. 2024). The two findings are better read as a range than a contradiction: lung adenocarcinoma brain metastases appear metabolically plastic, and the substrate a given lesion depends on is not predictable from primary histology alone. Direct *in vivo* tracing in primary human NSCLC shows that lactate is imported and oxidized as a tricarboxylic acid (TCA) carbon source, confirming that NSCLC can exploit circulating monocarboxylates as oxidative fuels (Faubert et al. 2017).

In SCLC, the clinical metabolic signal is most evident in lactate dehydrogenase (LDH). Elevated serum LDH is an independent predictor of poor survival in SCLC patients with brain metastases treated with whole-brain radiotherapy, consistent with LDH serving as a circulating proxy for glycolytic tumor burden (Anami et al. 2019). Serum metabolomics in newly diagnosed SCLC patients additionally shows elevated 3-hydroxybutyric acid and acetone (Pedersen et al. 2021), implicating ketone body metabolism among the deranged systemic programs at diagnosis, though whether this translates into ketone utilization within established brain lesions is unknown.

### Melanoma

Melanoma lesions concentrate in the frontal lobes (*∼*43%) with cerebellar lesions making up *∼*5% (Schroeder et al. 2020, Barrios et al. 2025). Multi-institutional atlas data and single-center mapping studies show that, compared with lung and breast primaries, melanoma is markedly less cerebellar predominant (Barrios et al. 2025). Clinically, melanoma brain metastases also show a high predisposition to hemorrhage, consistent with a vascular co-option strategy that weakens existing vessels rather than building new ones (Bentolila et al. 2016).

The metabolic program of MBM is dominated by oxidative phosphorylation and glutaminolysis. RNA-seq of surgically resected MBMs identifies an oxidative phosphorylation-enriched transcriptional state with elevated mTORC1 activity (Fischer et al. 2021). In a high oxidative phosphorylation MBM-derived model, metabolomics shows elevated 3-phosphoglycerate alongside glutamic acid and asparagine, and glutaminase inhibition reduces oxygen consumption rate without affecting extracellular acidification, consistent with glutamine-fueled oxidative phosphorylation coexisting with maintained glycolytic flux (Fischer et al. 2021). Layered onto this is a distinctive ketone body biology specific to BRAF V600E melanoma, which upregulates the ketogenic enzymatic activity, producing acetoacetate that enhances BRAF V600E binding to MEK1 and amplifies MEK/ERK signaling. *In vivo*, high fat dietary conditions that raise circulating acetoacetate (but not *β*-hydroxybutyrate) selectively promote growth of BRAF V600E xenografts (Xia et al. 2017). Pharmacologic blockade of acetoacetate-BRAF V600E binding with the inhibitory homologue dehydroacetic acid attenuates BRAF V600E tumor growth (Xia et al. 2017). In this subset of MBMs, there is a distinct shift from a glutamine-fueled to a ketone-fueled phenotype, establishing a causal, genotype-specific, and ketone-species-specific signaling axis in melanoma, and we return to its therapeutic implications in Section Therapeutic interventions: state of the art and pitfalls.

## Therapeutic interventions: state of the art and pitfalls

### Local therapies: vascular and structural interventions

The mainstays of local control, surgery, stereotactic radiosurgery (SRS), and whole brain radiation therapy (WBRT), target a metastasis by its size and location rather than its biology. Classically, surgical resection plus WBRT improved median overall survival to 9–10 months versus 3–6 months for WBRT alone (Patchell et al. 1990). Gross total resection is associated with longer survival than subtotal resection (Lin and DeAngelis 2015, Mills et al. 2022). Postoperative cavity SRS reduces 12-month local recurrence from 72% to 43% versus observation (Hatiboglu et al. 2020) and is increasingly preferred over postoperative WBRT for cognitive preservation, though it carries higher leptomeningeal disease and radionecrosis risks (Franchino et al. 2018). WBRT now plays a salvage and diffuse-disease role, with hippocampal-avoidance WBRT and concurrent memantine providing partial neuroprotection (Lin and DeAngelis 2015, Rishi and Yu 2020).

Each local modality is essentially blind to the metabolic phenotype of the lesion it treats. SRS dose is set by lesion size and proximity to critical structures, not by glycolytic dependence, oxidative phosphorylation dependence, or substrate availability. A small but suggestive body of evidence hints that radiosurgical response does differ by primary cancer in a direction loosely consistent with metabolic phenotype. In NSCLC, SRS achieves high short-term local response, with one series reporting complete or partial response in roughly three-quarters of treated lesions one to three months after treatment and no lesions progressing in that window (Won et al. 2015). BCBM, by contrast, appear less uniformly responsive, where a treatment-naive SRS cohort found that one-year local control was highest for NSCLC, intermediate for breast, and lowest for melanoma, with melanoma and breast converging by 2 years (Ene et al. 2024). There is, however, at least one metabolic signal already actionable at the level of radiation strategy. In the prophylactic setting for SCLC, high serum LDH identifies patients at greater risk of intracranial progression and worse survival (Anami et al. 2019), suggesting that a circulating metabolic marker could in principle help select patients for, or intensify, cranial radiation, and motivating the study of metabolic adjuncts even where local control is otherwise achievable. A metabolic adjunct has in fact already been tested in this setting, with oral L-arginine given before each radiation fraction lowering tumor lactate and improving control of brain metastases in a placebo-controlled trial (Marullo et al. 2021).

### Systemic therapies: signaling and indirect metabolic interventions

The past decade has produced systemic agents with substantial intracranial activity, giving options for local management of brain metastases. For EGFR-mutant NSCLC, the third-generation tyrosine kinase inhibitor (TKI) osimertinib produces a CNS objective response rate of *∼*91% in the phase 3 FLAURA trial versus 68% with first-generation TKIs, with fewer new CNS lesions, and longer overall survival (Soffietti et al. 2020). Similarly, for ALK-rearranged NSCLC, alectinib, brigatinib, and lorlatinib reduce CNS progression risk (Soffietti et al. 2020, Nardone et al. 2023). These agents now often permit deferral of upfront brain radiotherapy (Soffietti et al. 2020). For HER2+ breast cancer, tucatinib plus trastuzumab, and capecitabine in the HER2CLIMB trial improves progression-free survival (Galanti et al. 2021). For melanoma, nivolumab plus ipilimumab in the phase 2 trial CheckMate 204 produced intracranial clinical benefit and also improve progression-free survival (Achrol et al. 2019). BRAF/MEK inhibition achieves intracranial responses but with shorter durability, making it most useful for symptomatic patients needing rapid disease control (Achrol et al. 2019, Rishi and Yu 2020).

These regimens are signaling axis interventions, but their intracranial efficacy is shaped by metabolic and immune microenvironments. Immune checkpoint inhibitors act directly on the immune axis, but some notable failures to provide control in brain metastases are metabolic. For example, lactate accumulation in the tumor microenvironment suppresses T-cell and NK-cell effector function and stabilizes immunosuppressive myeloid phenotypes (Brand et al. 2016, Yi et al. 2025). Steroid-requiring symptomatic MBM and BCBM patients had a poor response to immune checkpoint inhibition (Soffietti et al. 2020), consistent with steroid-induced metabolic shifts potentiating tumor metabolic immunosuppression. Similarly in glioblastoma, although a primary tumor and not a metastatic lesion, concurrent dexamethasone reduced the benefit of PD-1 blockade in a dose-dependent manner in both syngeneic models and a clinical cohort (Iorgulescu et al. 2021). Because steroids are near-universal in symptomatic disease, the metabolic state of a lesion at the time of any intervention is partly iatrogenic, as corticosteroids also induce systemic hyperglycemia and insulin resistance. The strong signal of SRS plus the checkpoint inhibitor nivolumab in melanoma (Rishi and Yu 2020) suggests that local disruption of the BBB enables a more effective immune intervention, though the metabolic effects are as yet unclear. Beyond the tumor itself, fecal microbiota transplantation combined with immunotherapy has shown early activity in NSCLC and melanoma, hinting that the host metabolic and microbial context can be engineered to support intracranial immune responses (Duttagupta et al. 2026).

### The ketogenic diet paradox in melanoma: a worked example for personalization

The melanoma ketogenic diet literature provides the cleanest illustration of why metabolic interventions cannot be applied as blanket strategies, although we note that this evidence is experimental and not clinically validated as of yet. Two well conducted experimental studies predict opposite outcomes from the same intervention. In an immunocompetent metastatic melanoma model, ketogenic diet reduced blood glucose, elevated *β*-hydroxybutyrate, and was associated with fewer lung metastases (Weber et al. 2022). In their xenograft systems, neither *β*-hydroxybutyrate nor acetoacetate exerted a consistent direct proliferative effect, and the authors concluded that the anti-tumor effect of ketogenic diet was mediated by systemic and immune mechanisms rather than direct tumor-cell signaling (Weber et al. 2022). Conversely, in BRAF V600E melanoma, dietary fat-induced ketogenesis specifically elevates serum acetoacetate, directly potentiating BRAF V600E-MEK1 binding and ERK signaling, accelerating tumor growth *in vivo* (Xia et al. 2017). The same metabolic intervention is therefore predicted to be tumor-suppressive in BRAF wild-type melanoma but tumor-promoting in BRAF V600E disease. Because BRAF V600E occurs in roughly half of metastatic melanomas, the population-average effect of ketogenic diet on melanoma is essentially uninterpretable without genotype stratification.

The same logic generalizes to other metastatic tumors. In HER2+ BCBM with site-specific FASN dependence and a documented FABP7 program (Cordero et al. 2019, Ferraro et al. 2021), a high-fat dietary intervention could in principle either feed the lesion or, by overwhelming the FASN-dependent supply chain with exogenous lipid, perturb it. The right experiment has not been done to elucidate which populations can benefit or be harmed by these interventions. In SCLC, where serum ketone bodies are elevated at diagnosis (Pedersen et al. 2021) and serum LDH tracks intracranial relapse and survival (Anami et al. 2019), the systemic metabolic context for any dietary intervention is already abnormal. In glucose-independent brain-adapted breast cancer that oxidizes glutamine, glucose restriction is unlikely to provide selective pressure at all (Chen et al. 2024).

We note a parallel and complementary rationale for ketogenic intervention specific to brain metastasis. As mentioned before, ketosis is an anticonvulsant state, and seizures are a major source of morbidity and end-of-life mortality in brain tumor patients. Seizure incidence is as high as 73% in the 45 days before death, and perioperative seizures independently predict overall mortality (Brauchitsch et al. 2022, Zheng et al. 2022). Cortical lesions carry higher seizure risk than deep ones (Wolpert et al. 2020), which places melanoma (frontal-predominant) and triple-negative breast cancer (frontal, parietal, and limbic) at particular risk. Even where ketogenic diet’s direct antitumor effect is uncertain or unfavorable, its anti-epileptic effect may justify its use in selected patients.

## The future of targeted metabolic therapeutics

A personalized approach to the metabolic management of brain metastases will require advances on three fronts: matching metabolic interventions to tumor genotype and lesion location, predicting metabolic phenotype before lesions are established, and modeling how metabolic vulnerability shifts over time and with systemic therapy.

### Predicting brain metastatic metabolic phenotype from the primary tumor and first metastasis

The clearest near-term opportunity is to match a metabolic intervention to a lesion’s known dependency rather than applying it uniformly. The most compelling recent proof of concept comes from MYCN-driven neuroblastoma, where an arginine- and proline-free diet was shown to deplete the polyamine precursor ornithine and potentiate the ornithine decarboxylase inhibitor difluoromethylornithine, reprogramming translation to suppress proliferation and drive differentiation (Cherkaoui et al. 2025). The principle is that a precisely specified diet, engineered against a genotype-driven metabolic dependency and paired with a targeted drug, can potentiate a large and durable effect that neither achieves alone. When a brain metastasis’s metabolic requirement is known, the intervention can in principle be designed to match it: glutaminase inhibition with CB-839 has direct preclinical support in oxidative phosphorylation-high, glutamine-dependent melanoma brain metastases (Fischer et al. 2021); the site-specific FASN dependence of HER2+ BCBM nominates FASN inhibition as a candidate selective for intracranial disease (Ferraro et al. 2021); and the glycolytic, lactate-exporting phenotype of much lung-derived disease motivates LDH or monocarboxylate-transporter inhibition, supported both by the lung brain-metastasis glycolysis literature (Yi et al. 2025) and by serum LDH’s prognostic signal in SCLC (Anami et al. 2019).

Metabolic plasticity is the principal barrier to any of these. Brain-adapted breast cancer cells can grow glucose-independently on glutamine and branched-chain amino acids (Chen et al. 2015), which is precisely an escape from glucose restriction; lung adenocarcinoma spans glycolytic and oxidative states (Faubert et al. 2017, Duan et al. 2024); and metabolic heterogeneity increases as metastases progress (Faubert et al. 2020). A single-substrate restriction applied alone should therefore be expected to select for the escape route rather than to control disease. This is the argument for pairing rather than substituting: the neuroblastoma result potentially worked because the diet and the drug closed the same pathway from two directions (Cherkaoui et al. 2025), and the same logic would apply to combining glucose restriction with glutaminase inhibition in disease that has both options available.

The ketogenic diet sits within this scheme because its effect is phenotype-dependent. It is plausibly contraindicated where it would raise acetoacetate in BRAF V600E melanoma, plausibly beneficial in BRAF wild-type disease, and of uncertain value in tumors that have abandoned glucose dependence altogether. Even a low-cost intervention is therefore not universally safe. Additionally, hyperglycemia is an adverse prognostic factor in brain metastases treated with radiotherapy, raising the possibility that glucose control is a cheap and broadly applicable adjunct, though this remains to be tested prospectively in the brain metastasis setting (Poudyal et al. 2025). The unifying prescription is that “metabolic therapy” is not one intervention but a family of them, and selecting among them requires knowing which dependency a given lesion actually has.

### Longitudinal modeling of metastatic potential and regional vulnerability

Metabolic reprogramming is characteristic of tumor progression, and as metastases progress their metabolic heterogeneity increases (Faubert et al. 2020), which is likely to render single-axis interventions insufficient for established disease. The implication is that the window for targeted metabolic therapy is early, before metabolic divergence accumulates, and that screening sensitive enough to detect early metastatic disease, combined with metabolic phenotyping of those small lesions, would be most valuable. Longitudinal imaging that integrates anatomic localization, perfusion (the vascular axis), metabolic imaging such as FDG-PET and hyperpolarized 13C-pyruvate, and immune with metabolic measurements such as MRSI (Lucchetti et al. 2025) would supply the empirical substrate for dynamic models of regional vulnerability that update over time. Computational frameworks that represent tumor metabolism along explicit catabolic–anabolic axes are beginning to make such dynamic modeling tractable, offering a route from static snapshots toward forecasts of how a given lesion’s vulnerabilities will move (Villela-Castrejon et al. 2025). Methods developed for modeling complex neural dynamics are transferable to this problem: principled model-reduction approaches that distill tractable predictive models from high-dimensional data (Antal et al. 2024) address the central difficulty of forecasting metabolic divergence, and the broader precision-neurotherapeutics agenda reshaping neurologic and psychiatric intervention (Mujica-Parodi et al. 2026) extends naturally to metabolic targeting in brain metastasis. The same probabilistic frameworks already applied to function-sparing palliative radiation could be extended to forecast where in the brain new lesions are most likely to appear given a patient’s primary tumor and current treatment state.

### Coupling systemic and cellular metabolic states

The interventions discussed so far act on the tumor cell or its immediate niche, but a tumor cell’s metabolic options are bounded by the systemic state of the patient, which is also modifiable. Two lines of evidence, while none yet brain-metastasis-specific, suggest that whole-body metabolic context will need to be part of the personalization. First, diet beyond ketosis impacts tumor metabolism, immunity, and the microbiome simultaneously. A high-fiber, plant-based dietary intervention in myeloma precursor disease altered body mass, insulin resistance, microbiome composition, and inflammatory monocyte subsets together (Shah et al. 2026), illustrating that dietary intervention effects on a metabolically defined tumor may be mediated as much through host immunity and the gut as through substrate supply. Dietary changes can also target other organs that are sensitive to ketogenic flux and are common sites of metastasis, such as the liver. Short-term ketogenic intervention has been shown to reduce intrahepatic triglycerides in humans (Luukkonen et al. 2020), and can provide support to lipogenic-dependent metabolism when the liver requires it (Chen et al. 2024). Second, structured exercise has moved from a supportive to disease-modifying intervention. A randomized trial in resected colon cancer showed that a structured exercise program after adjuvant chemotherapy reduced recurrence and improved survival (Courneya et al. 2025). Mechanistic literature attributes such effects to coordinated changes in inflammation, growth factor signaling, immune surveillance, and epigenetic state (Amirsasan et al. 2022). Exercise also mitigates treatment toxicity, including chemotherapy-associated cognitive impairment (Mustian et al. 2026), particularly relevant when the disease and its treatments both threaten neurocognition. Because different exercise modalities elicit distinct systemic metabolic responses, exercise is plausibly a tunable metabolic intervention rather than a generic health measure.

The unifying theme is that cellular metabolic state cannot be interpreted in isolation from the organism’s metabolic state. A targeted metabolic therapy chosen correctly for a lesion’s genotype may still succeed or fail depending on the patient’s diet and activity. Bringing metabolism into precision brain metastasis care will ultimately mean modeling the tumor cell and the host as a single coupled system.

## Conclusion

Brain metastases are not uniformly distributed in the brain in part because the brain is not metabolically, vascularly, or immunologically uniform. We propose that different primary cancers seek different regions because their intrinsic metabolic and signaling programs interact differently with regional vascular caliber, baseline metabolism, glia-neuron composition, and immune architecture. Of the three axes that define the metastatic niche, metabolism is the least exploited clinically and the one whose interventions most clearly demonstrate that one-size-fits-all is the wrong frame. The melanoma ketogenic diet paradox, in which the same intervention is predicted to suppress BRAF wild-type and accelerate BRAF V600E melanoma, captures this in miniature. The path forward is to integrate metabolic phenotyping with the molecular and anatomic information already used for therapy selection, to forecast brain metastatic metabolic phenotype from primary tumor profiling, and to track metabolic divergence longitudinally, while remembering that the tumor cell’s metabolism is embedded in, and constrained by, the metabolic state of the whole patient. Local therapies, the dominant systemic regimens, and metabolic interventions all stand to benefit from being chosen with the niche and the host in mind.
